# Histomorphological variations in progressive multifocal leukoencephalopathy correlated with JCV replication in brain lesions: insights from 91 patients

**DOI:** 10.1186/s40478-025-02027-7

**Published:** 2025-05-19

**Authors:** Kenta Takahashi, Yuko Sato, Hideki Hasegawa, Harutaka Katano, Tadaki Suzuki

**Affiliations:** 1 Department of Infectious Disease Pathology, National Institute of Infectious Diseases, Japan Institute for Health Security, Shinjuku, Tokyo Japan; 2 Influenza Research Center, National Institute of Infectious Diseases, Japan Institute for Health Security, Musashimurayama, Tokyo Japan; 3 Department of Infectious Disease Pathobiology, Graduate School of Medicine, Chiba University, Chiba, Japan

**Keywords:** Progressive multifocal leukoencephalopathy, JC polyomavirus, Brain biopsy, Histopathology, Immunohistochemistry, Tissue-based PCR

## Abstract

Progressive multifocal leukoencephalopathy (PML) is a demyelinating disease caused by JC polyomavirus (JCV). The histopathology of PML is morphologically diverse and characterized by the classical triad of demyelination, enlarged oligodendroglial nuclei, and bizarre astrocytes. Pathological diagnostic criteria for PML require both the classical triad and viral detection in brain tissue. However, the frequency of this triad in surgical pathology specimens and its correlation with disease progression and viral loads remain unclear. In this study, 117 brain tissues from 91 pathologically confirmed PML patients were investigated. PML histopathology was found to be spatially and temporally pleomorphic, and not all brain tissues exhibited the complete classical triad. The sensitivity of quantitative PCR for detecting JCV in brain tissues was 100%, whereas that of immunohistochemistry (IHC) was 83.5–87.8%. Viral loads in biopsy samples were significantly higher than those in autopsy samples and decreased over time after disease onset. To systematically characterize PML lesions from the outer border to the demyelinated center, we developed a histological classification based on the classical triad and macrophage infiltration. This classification correlated with viral loads, with subtypes characterized by abundant enlarged oligodendroglial nuclei at the demyelination border exhibiting the highest levels of JCV DNA. Pathological variability was influenced by spatial and temporal factors rather than by underlying diseases, although PML associated with acquired immunodeficiency syndrome exhibited more severe demyelination. In conclusion, histomorphological variability in PML reflects viral replication activity, emphasizing the importance of comprehensive pathological evaluation. Combining histomorphology, tissue-based PCR for viral DNA detection, and IHC for viral antigens is crucial for assessing disease progression. Early brain biopsy from the demyelination border offers the best opportunity for a definitive diagnosis of PML and may guide therapy targeting active lesions.

**Supplementary Information:**

The online version contains supplementary material available at 10.1186/s40478-025-02027-7.

## Introduction

Progressive multifocal leukoencephalopathy (PML) is a rare but often fatal demyelinating disease caused by opportunistic infection with JC polyomavirus (JCPyV, JCV) [1, 2]. It primarily affects immunosuppressed individuals, including those with acquired immunodeficiency syndrome (AIDS), hematologic malignancies, autoimmune diseases, and solid organ transplant recipients [1, 3–7]. Recently, PML has also been reported in patients receiving monoclonal antibodies or small-molecule immunomodulators such as natalizumab and fingolimod [8–11].

Histopathological examination of brain tissue is crucial for a definite diagnosis of PML, along with the detection of JCV DNA in cerebrospinal fluid (CSF) via polymerase chain reaction (PCR). Pathologically definite PML requires both histomorphological evaluation and the detection of JCV in brain tissues [2, 12]. The classical histomorphological triad of PML consists of demyelination, enlarged oligodendroglial nuclei and bizarre astrocytes [12, 13]. In addition to histomorphological findings, electron microscopy, immunohistochemistry (IHC), and PCR are used to detect JCV particles, proteins, and DNA in brain tissue samples [12].

PML pathology exhibits significant spatial and temporal variability [14, 15]. Enlarged oligodendroglial nuclei typically outline the margins of demyelination foci [16], but these features can be difficult to detect in advanced, so-called ‘‘burnt-out’’ cases of PML [17, 18]. Large bizarre astrocytes are commonly found in the central areas of expanding demyelinated regions [16]. Previous studies have shown that the distribution and quantity of JC virus-infected oligodendrocytes and infiltrating macrophages within demyelinating lesions can vary [15]. In particular, PML in AIDS patients has been associated with more severe tissue damage [16]. As a result, pathological findings may differ depending on the sampling location, disease progression, and underlying conditions. Moreover, although the classical histomorphological triad is described in the PML diagnostic criteria [12], it is not always detectable in pathological brain tissue samples, especially in small biopsy samples [19]. The frequency of observing the full triad in large case series remains uncertain, and the necessity of identifying all three elements is not fully understood. Additionally, there is limited evidence regarding the relationship between PML histomorphology and JC viral replication activity, particularly when different underlying diseases are considered.

Real-time PCR allows highly sensitive quantification of JCV DNA, with copy numbers directly reflecting viral replication activity [20–22]. However, while real-time PCR provides accurate quantification, it does not offer information on the viral distribution or localization within the tissue. The pathological diagnostic criterion for PML includes the detection of JCV DNA via tissue-based PCR [12]. The sensitivity of nested PCR in PML brain tissue samples has been reported to be 100%, and real-time PCR for JCV DNA in cerebrospinal fluid (CSF) can detect as few as 10 copies/ml with 100% specificity [23, 24]. However, quantitative data on viral DNA loads in large case series of PML brain tissues are lacking, and the relationships among the viral DNA load, histomorphological characteristics, disease progression, and time from PML onset to tissue sampling remain unclear.

IHC is another essential method for the pathological examination of PML brain tissues [12]. While IHC is useful for evaluating the presence and distribution of viral proteins in tissue, it is a qualitative method and does not allow for precise quantification of viral loads. Although a comparison of JCV IHC positivity with JCV DNA-labeled cells by in situ hybridization (ISH) has been reported [25], there is limited evidence on the relationships among IHC positivity, viral DNA copy numbers and PML disease progression. The absence of comprehensive descriptions of the dynamic histomorphological changes in PML tissues complicates accurate diagnosis and the development of individualized treatment strategies.

In this study, a total of 117 brain samples from 91 patients with pathologically confirmed PML and various underlying diseases were investigated. We established an easy-to-use scoring system of histomorphological characteristics for routine histological evaluation and clarified the frequency of the classical morphological triad in PML brain tissue samples. Using tissue-based quantitative PCR for JCV DNA and IHC for JCV antigens, we investigated the relationship between viral replication activity and histomorphological characteristics. Our findings suggest a strong correlation between viral replication and the histomorphological features of PML brains, emphasizing the importance of selecting appropriate biopsy sites and timing for accurate diagnosis and individualized antiviral therapy.

## Materials and methods

### Clinical samples

A total of 117 brain tissue samples, consisting of 75 biopsies and 42 autopsies from 91 patients (male = 58.4%, mean age = 60.9 ± 14.0 years), were examined. All the samples were histopathologically diagnosed with PML. The underlying conditions of the PML patients included hematologic malignancies (29 patients), autoimmune diseases (19 patients), AIDS (14 patients), solid organ transplantation (5 patients), renal dysfunction (5 patients), solid organ cancer (5 patients), acquired immunodeficiency excluding AIDS (4 patients), sarcoidosis (4 patients), congenital immunodeficiency (2 patients), hepatic dysfunction (2 patients), no obvious underlying diseases (5 patients), and unknown conditions (2 patients). Five patients had overlapping conditions: two had hematologic malignancies with autoimmune disease, one had hematologic malignancy and solid organ cancer, one had solid organ cancer and sarcoidosis, and one had both renal and hepatic dysfunction. Biopsy samples were obtained from brain regions showing abnormalities on MRI or other imaging studies for histological diagnosis. Most biopsy cases consisted of a single specimen per patient; however, when multiple samples were clinically obtained, up to three tissues were analyzed. In autopsy cases, a comprehensive pathological examination of the entire brain was performed, and up to seven available sections per patient containing lesions were selected for analysis. All personal information was anonymized by the hospital or institutions that provided the samples, and the NIID received the specimens labeled only with anonymous IDs. The samples included formalin-fixed paraffin-embedded (FFPE) tissues and/or frozen samples.

### Scoring system for histomorphological evaluation

Hematoxylin and eosin (HE) staining was performed on FFPE sections following standard protocols. HE-stained sections and Klüver Barrera (KB) double-stained slides, combined with neurofilament immunohistochemistry (NF-IHC), were evaluated microscopically. The morphological features of PML, including the pathological triad of demyelination, enlarged oligodendroglial nuclei, and bizarre astrocytes, as well as atypical astrocytes and infiltration of myelin-laden foamy macrophages, were assessed and scored on the basis of their severity or density across each whole slide. Demyelination was scored via double-stained KB and NF-IHC (KB-NF), with scores ranging from 0 to 3:

- Score 0: Not obvious.
- Score 1: Mild, focal demyelinating lesion visible only with KB-NF.
- Score 2: Moderate, characterized by paler neural parenchyma on HE and a lack of a myelin sheath but with preserved axons with KB-NF.
- Score 3: Severe, with a fully demyelinated lesion with axonal damage or loss.

Enlarged oligodendroglial nuclei, bizarre astrocytes, atypical astrocytes, and foamy macrophage infiltration were scored on HE-stained slides. Bizarre astrocytes were distinguished from atypical astrocytes by their multinucleated morphology and/or giant cell size. These features were scored from 0 to 3:

- Score 0: None or not obvious.
- Score 1: Mild, with only a few cells present.
- Score 2: Moderate, easily detected at low-power field (LPF) but not abundant in high-power field (HPF).
- Score 3: Severe, with numerous cells or dense foamy macrophage infiltration in the HPF.

**Fig. 1:**
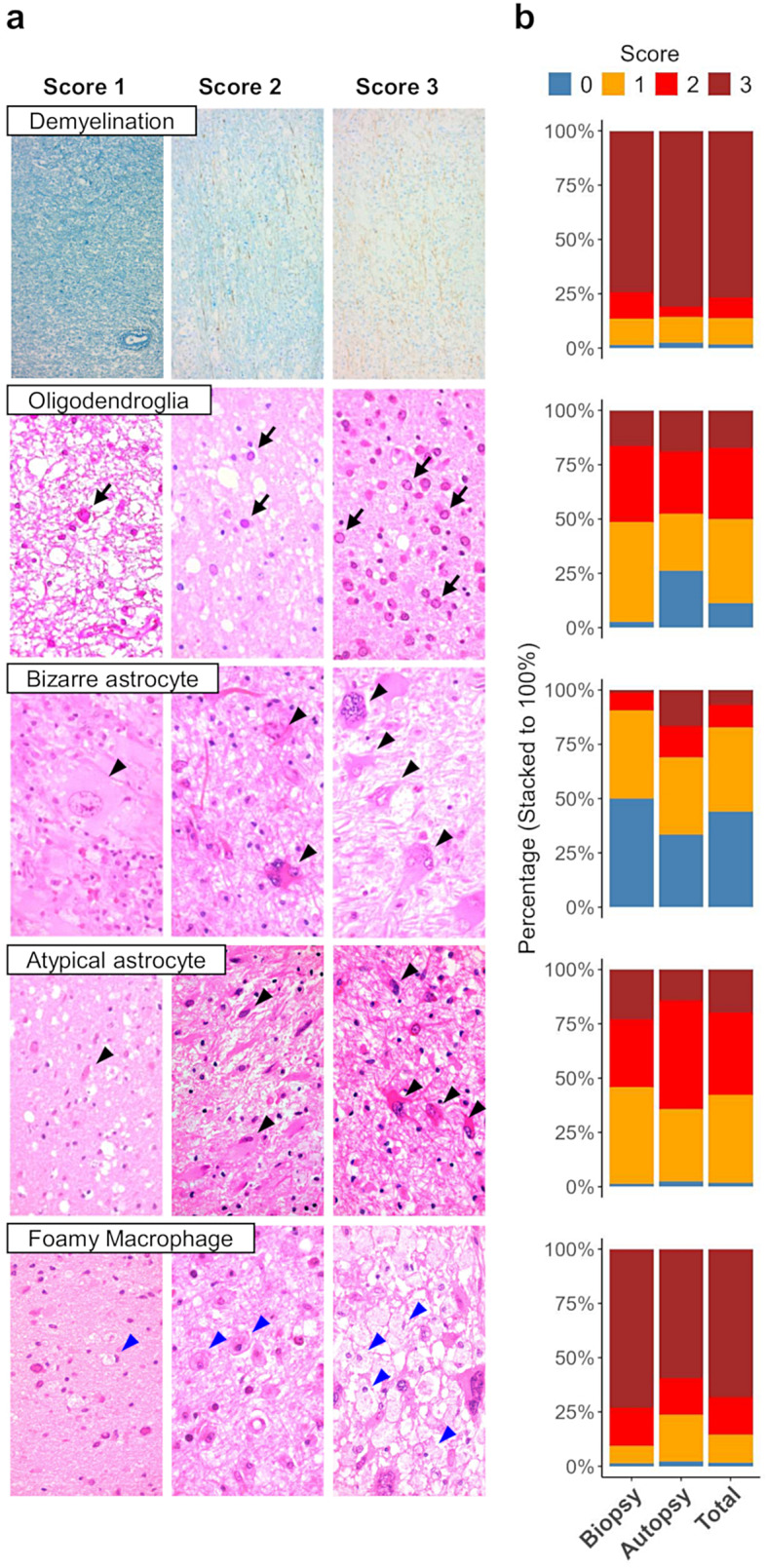
Scoring of PML morphological features and their frequency in the samples. **a** PML morphological features and corresponding scores (scores of 1–3) were as follows: demyelination, enlarged oligodendroglial nuclei, bizarre astrocytes, atypical astrocytes and foamy macrophage infiltration. Demyelination was evaluated by Klüver Barrera double-staining with neurofilament immunohistochemistry. Other features were evaluated via HE staining. Arrows and arrowheads indicate representative cells. **b** Frequency distribution of each morphological feature score in biopsy (*n* = 74), autopsy (*n* = 42), and total samples (*n* = 116). A score of 0 indicates that no PML features were observed

Representative fields are shown in Fig. 1a.

### DNA and RNA extraction

DNA was extracted from FFPE or frozen brain samples via the QIAamp DNA FFPE Tissue Kit or DNeasy Blood and Tissue Kit (Qiagen, Hilden, Germany), respectively.

### Real-time PCR

Duplex real-time PCR for JC virus DNA and beta-actin was performed to confirm the diagnosis of PML and quantify the viral load in brain samples, following the standard TaqMan™ protocol on an Agilent Mx3000P. The 20 µL reaction mixture contained 1x Thunderbird Probe qPCR Mix (Toyobo), 0.3 µM TaqMan™ probe, 0.3 µM forward and reverse primers, and 1 µL of template. The reactions were incubated at 95 °C for 15 min, followed by 40 cycles of 95 °C for 15 s and 60 °C for 1 min. Probes and primer sequences were reported previously [26, 27]. JCV genome copy numbers per cell were calculated by dividing the viral copy numbers by half of the copy numbers of beta-actin, as each cell had two copies of the endogenous gene in two alleles [20].

### Immunohistochemistry (IHC)

FFPE sections of PML brains were deparaffinized with xylene, dehydrated with ethanol, and treated with citric acid buffer (pH 6.0) at 121 °C for 10 min to quench endogenous activity. The sections were then incubated with primary antibodies and stained using an EnVision™ kit (Dako Cytomation, Copenhagen, Denmark). The primary antibodies used were as follows: neurofilament (mouse monoclonal, clone 2F11, Agilent, Santa Clara, Calif., USA); SV40 large T antigen (TAg, mouse monoclonal, clone PAb416b, Calbiochem, San Diego, Calif., USA), which has been confirmed to cross-react with JCV TAg [28]; and rabbit polyclonal antibodies against JCV VP1, VP2/3 and JCV agnoprotein [29–31].

### Scoring system of immunohistochemistry for viral proteins

All the IHC-stained slides were incubated with antibodies against JC viral proteins and scored according to the number of immunohistochemically positive cells. The entire sample was first examined at LPF, and the area with the highest density of positive cells was selected. Positive cells were then counted in 5 HPFs, and scores were assigned as follows:

- Score 0: No positive cells.
- Score 1: 1 to < 5 positive cells in 5 HPFs.
- Score 2: 5 to < 50 positive cells in 5 HPFs.
- Score 3: 50 to < 500 positive cells in 5 HPFs.
- Score 4: ≥ 500 positive cells in 5 HPFs.

Representative fields are shown in Fig. 7a.

**Fig. 2:**
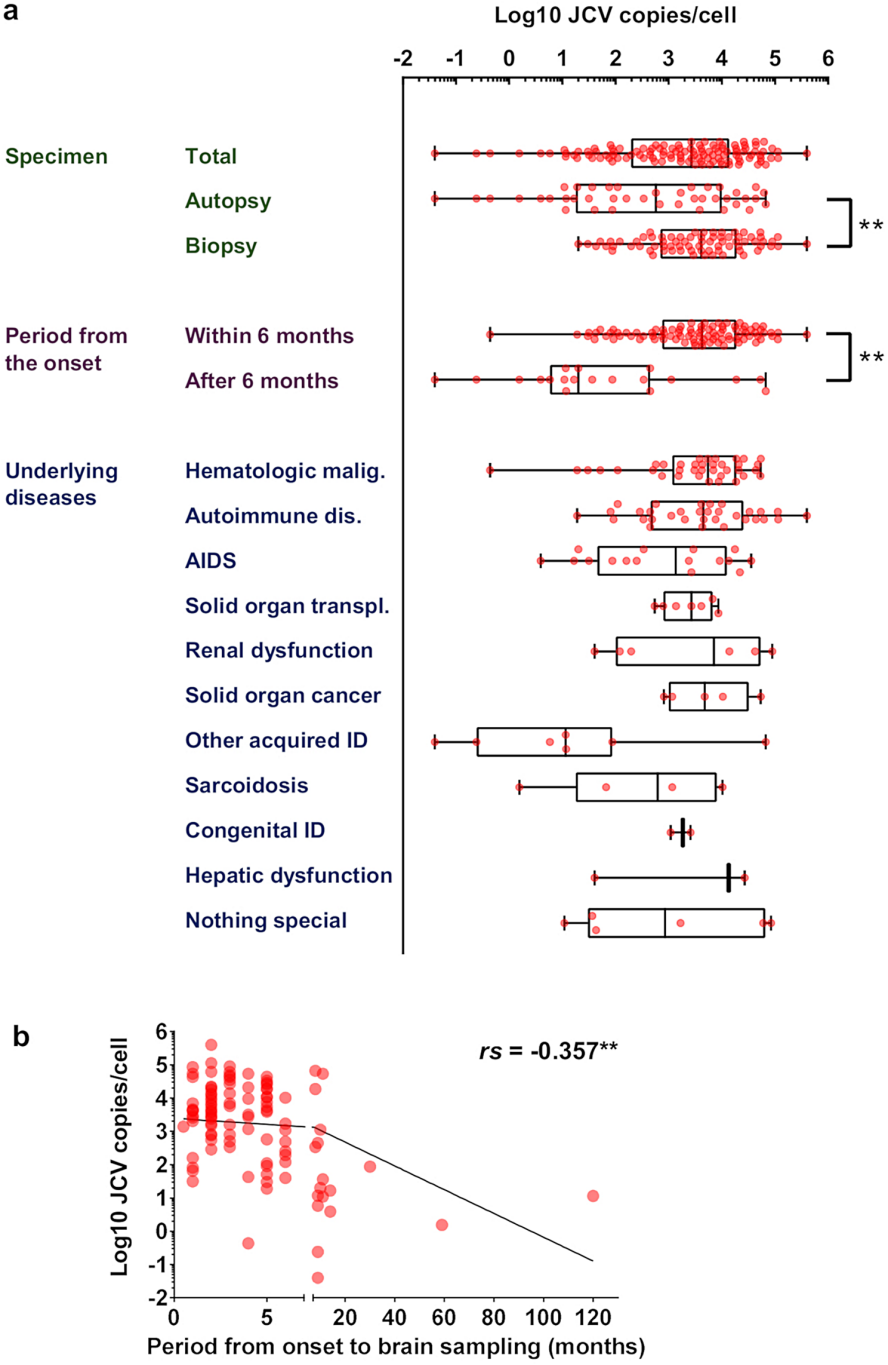
Quantification of JCV DNA in PML brain tissues. **a** JCV copies in PML brain tissues. Autopsy and biopsy samples (*n* = 41 and 74), tissues sampled within 6 months of symptom onset or later (*n* = 91 and 19), and specimens from patients with each underlying disease were used. Overlapping underlying conditions are counted in each category (total *n* = 123). Boxes show medians and 25% and 75% percentiles, and whiskers indicate the range from minimum to maximum values. Asterisks indicate significant differences between groups. **p < 0.01. ID: immunodeficiency. **b** Relationship between the period from symptom onset to brain sampling and JCV copies in the brain (*n* = 110). Fitted lines are shown with Spearman’s correlation coefficient (*rs*). **p < 0.01

### Statistics

Statistical normality was analyzed via D’Agostino and Pearson normality tests. Statistical differences were evaluated via the Mann‒Whitney U test or t test, and correlations were analyzed via Spearman’s rank correlation coefficient or the Pearson product‒moment correlation coefficient (GraphPad Prism 9, GraphPad Software, La Jolla, CA, USA). Statistical significance was set at *p* < 0.05.

## Results

### Histomorphological characteristics of PML brain tissues

Among the 117 brain tissue samples analyzed, 116 pathologically confirmed PML samples, including 42 autopsy samples and 74 biopsy samples, underwent morphological examination. One biopsy sample was excluded from morphological evaluation due to its unavailability of the original HE slide from the initial diagnosis. The classical histomorphological triad of PML was evaluated and scored from 0 to 3 (Fig. 1a). Demyelination, enlarged oligodendroglial nuclei, and bizarre astrocytes were observed in 98.3%, 88.8%, and 56.0% of the samples, respectively (Fig. 1b), indicating that the classical histomorphological triad was not always present in all cases. Similarly, atypical astrocyte and foamy macrophage infiltration were also assessed via the PML histomorphological scoring system (Fig. 1a), with both being observed in 98.3% of the samples (Fig. 1b). Notably, compared with biopsy cases, autopsy cases lacking enlarged oligodendroglial nuclei were relatively common (26.2%), and bizarre astrocytes were not observed in half of the biopsy cases (50.0%) (Fig. 1b). The PML histomorphological scores were also compared among the three major underlying diseases. The demyelination score in AIDS-related PML patients was significantly greater than that in patients with hematologic malignancies or autoimmune diseases (*p* = 0.043 and *p* < 0.01, respectively), whereas other histomorphological scores were not significantly different (Suppl. Fig. S1).

### Quantification of JCV DNA loads in PML brain tissues

To quantify JCV DNA loads in PML brain tissues, real-time PCR was performed using DNA extracted from the brain samples. Quantitative PCR was successfully conducted on 115 of the 117 pathologically diagnosed PML brain tissues (two samples had only slides available for histomorphological study). JCV DNA was detected in all 115 tissues, yielding 100% sensitivity for real-time PCR. The median and geometric mean JCV DNA copy numbers in PML brain tissues were 2,680 and 1,445 copies per cell, respectively (Fig. 2a; Table 1). Compared with the autopsy samples, the biopsy samples presented significantly greater viral DNA copy numbers (*n* = 74 and 41, median of 4,115 vs. 572 copies per cell, geometric mean of 3,171 vs. 350.1 copies per cell, *p* < 0.01) (Fig. 2a; Table 1), suggesting that the biopsy samples were taken from tissue during the period of more active viral replication than the autopsy samples were. In addition, among the 115 samples successfully analyzed, 14 were frozen samples, while the remaining 101 were FFPE samples. Since the overall JCV DNA copy numbers did not differ significantly between FFPE and frozen samples in this study (median of 3,850 vs. 496.5 copies per cell, *p* = 0.07, unpaired t-test, Suppl. Fig. S2a), we used FFPE results for cases where both FFPE and frozen tissues were available as FFPE sections reflected histological evaluation more directly. Moreover, to further validate this, we conducted a direct comparison of JCV DNA quantification between FFPE and frozen samples. Using 39 paired samples with both FFPE and frozen tissues available, we found that FFPE samples yielded significantly higher JCV DNA copy numbers than frozen samples (median of 4,570 vs. 2,530 copies per cell, geometric mean of 20,255 vs. 7,042 copies per cell, *p* < 0.01, paired t-test, Suppl. Fig. S2b). However, a significant correlation was observed between the results from FFPE and frozen samples (Pearson correlation coefficient (*r*) = 0.845, *p* < 0.01, Suppl. Fig. S2c). This difference is likely due to the fact that FFPE samples were specifically selected to contain JCV-positive lesions with morphological examination, whereas frozen samples were collected without prior histological confirmation of the affected tissue.

**Table 1:** Percentile, median, and geometric means of JCV genome copies in PML autopsy and biopsy brain samples

	Total	Autopsy	Biopsy
Sample number	115	41	74
25% Percentile	197	17.75	699
Median	2680	572	4115
75% Percentile	13,900	10,025	19,050
10% Percentile	19.6	2.012	89
90% Percentile	49,960	41,420	54,100
Geometric mean	1445	350.1	3171
Geometric SD factor	21.54	44.22	9.554
Lower 95% CI of geo. mean	819.8	105.9	1880
Upper 95% CI of geo. mean	2548	1158	5349

Viral DNA loads were also compared across the major underlying diseases: hematologic malignancy, autoimmune disease, and AIDS. The median and geometric mean viral DNA copy numbers were 5,460 and 2,926 (*n* = 37), 4,425 and 3,520 (*n* = 32), and 1,363 and 666.3 copies per cell (*n* = 16), respectively, with no significant differences observed across these underlying diseases. The sample sizes for other underlying health conditions were too small for analysis (Fig. 2a; Table 2).

**Table 2:** Percentile, median, and geometric means of JCV genome copies in PML brain samples according to underlying diseases

Underlying disease	Hematologic malignancy	Autoimmune disease	AIDS	Solid organ transplantation	Renal dysfunction	Solid organ cancer	Other acquired immunodeficiency	Sarcoidosis	Congenital immunodeficiency	Hepatic dysfunction	Nothing special
Sample number	37	32	16	7	6	5	7	4	2	2	6
25% Percentile	1165	457	45.33	785	100.8	991	0.24	17.68	1090	40.2	30.1
Median	5460	4425	1363	2680	7049	4750	11.6	618.1	1845	13,470	861.4
75% Percentile	18,650	25,800	12,413	6690	53,975	32,250	87	8018	2600	26,900	65,975
10% Percentile	47.3	97.47	12.72	546	40.2	812	0.04	1.54	1090	40.2	10.9
90% Percentile	44,360	97,670	25,910	8600	89,000	10,500	66,800	10,300	2600	26,900	84,500
Geometric mean	2926	3520	666.3	2333	1920	4804	12.38	187.1	1683	1040	724.4
Geometric SD factor	12.34	11.25	18.93	2.883	28.38	5.498	98.95	45.15	1.849	99.53	51.11
Lower 95% CI of geo. mean	1266	1471	139	876.2	57.34	578.7	0.1766	0.4358	6.724	1.164e-015	11.67
Upper 95% CI of geo. mean	6763	8425	3193	6210	64,309	39,878	867	80,372	421,496	9.286e + 020	44,971

The relationships between the time from symptom onset to brain sampling and the number of viral DNA copies were also analyzed. PML brains in the acute and subacute phases (within 6 months of onset) had significantly higher viral DNA copy numbers than those in the late phase (after 6 months of onset) (*n* = 91 and 19, median 4,230 vs. 20 copies per cell, geometric mean 2,979 vs. 57.41 copies per cell, *p* < 0.01) (Fig. 2a). Additionally, a significant negative correlation was found between viral DNA copy number and the time from symptom onset to brain sampling (Spearman *r*, *rs* = -0.357, *p* < 0.01) (Fig. 2b). These findings suggest that brain biopsy is more effective for detecting JCV within 6 months after symptom onset, whereas prolonged cases, particularly those lasting over a year, tend to have lower viral DNA loads.

### PML histomorphological features and JCV copies

**Fig. 3:**
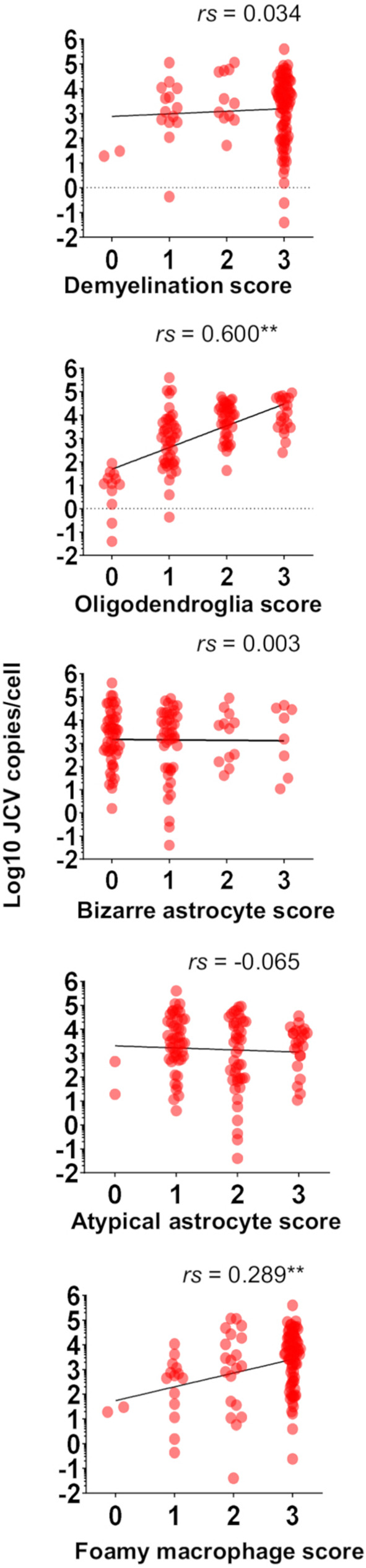
Correlation between PML morphological feature scores and JCV copies in brain tissue (*n* = 114). The dots indicate the copy number per cell in each sample. Fitted lines are shown with Spearman’s correlation coefficient (*rs*). **p < 0.01

The correlation between the PML histomorphological scores and JCV DNA copy numbers in the brain tissue was evaluated (Fig. 3; Table 3). Among the histomorphological features, enlarged oligodendroglial nuclei presented the strongest correlation with viral DNA copy numbers in brain tissue (*rs* = 0.600, *p* < 0.01), followed by foamy macrophage infiltration scores (*rs* = 0.289, *p* < 0.01) (Fig. 3). Other features, such as demyelination, bizarre astrocytes, and atypical astrocytes, did not exhibit significant correlations with the viral DNA load (Fig. 3).

**Table 3:** Correlation of PML morphological scores and JCV copies. Ns: not significant

HE Morphology	Spearman *r* (rs)	*p* value	Sample number
Demyelination	0.034	0.716 (ns)	114
Enlarged nucleated oligodendroglia	0.600	< 0.01	114
Bizarre astrocyte	0.003	0.976 (ns)	114
Atypical astrocyte	-0.065	0.492 (ns)	114
Foamy macrophage infiltration	0.289	< 0.01	114

### Classification of PML morphological types on the basis of histomorphological characteristics and JCV loads

**Fig. 4:**
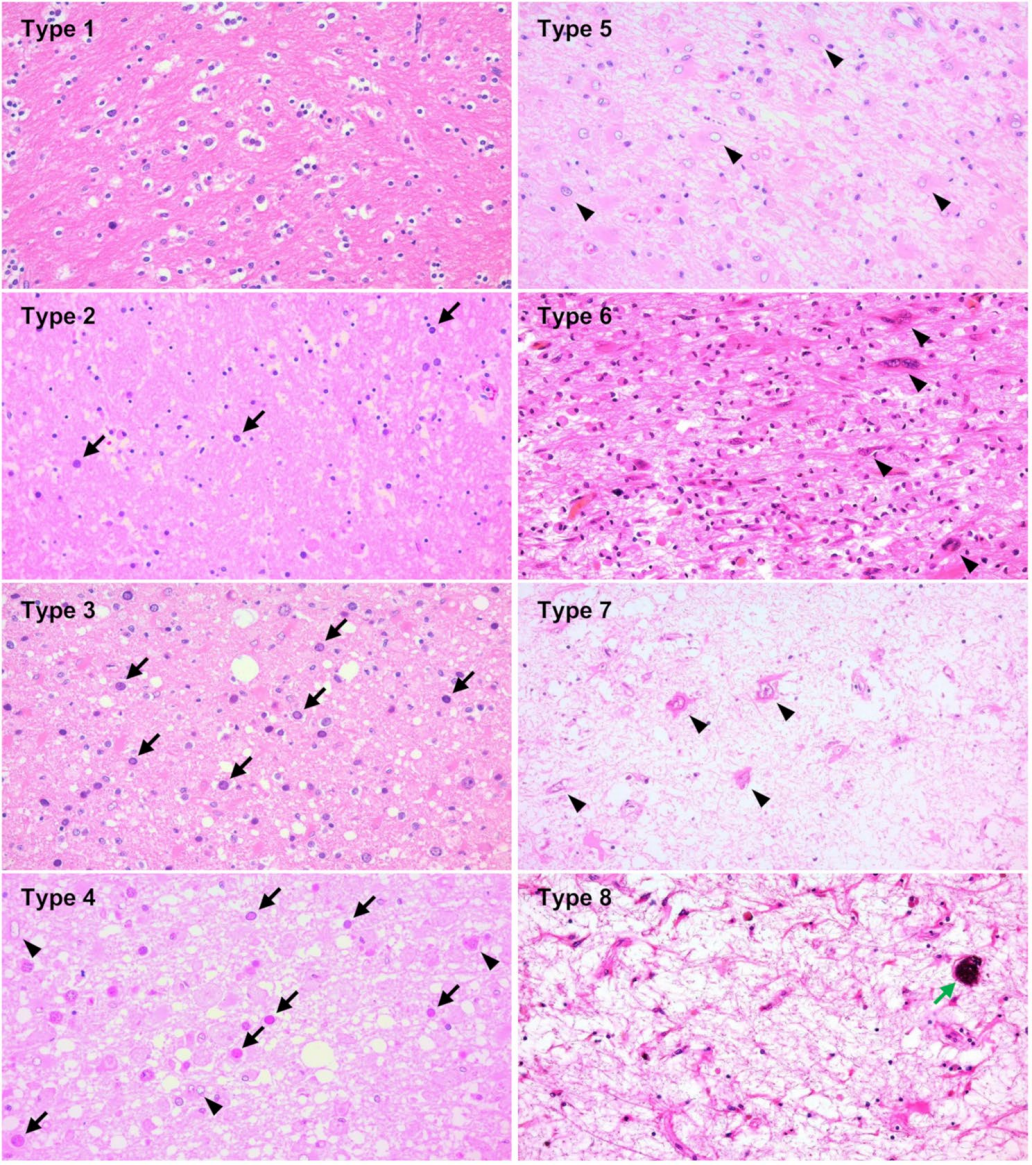
Histomorphological typing of PML lesions. Representative histological images of different PML morphological types were selected from various cases. Type 1 lesions, which are the outermost and adjacent to normal tissue, did not show any demyelination, with only slight histological changes observed via HE staining of the brain tissues. Type 2 lesions presented increased numbers of enlarged oligodendroglial nuclei (arrows) and the development of demyelination. The type 3 lesions presented numerous enlarged oligodendroglial nuclei (arrows), with demyelination continuing to progress. This process reached its peak in type 4 lesions (arrows), where tissue reactions were prominent. Reactive astrocytes were observed (arrowheads). In type 5 lesions, the demyelinated areas are filled with reactive or atypical astrocytes (arrowheads), and abundant foamy macrophages engage in myelin phagocytosis. Type 6 represented the stage where demyelination was most severe, and bizarre astrocytes (arrowheads) became the dominant feature. Type 7 lesions display sparse tissue with lingering bizarre astrocytes (arrowheads) but little to no reaction to complete demyelination. Finally, Type 8, the terminal stage of PML lesions, presented as a “burnt-out” lesion with low cellularity and occasional calcification (green arrow)

PML lesions exhibit a broad spectrum of histomorphological variability, particularly in autopsy samples, where the histology of lesions changes sequentially from the outer border to the demyelinated center [14, 15]. This histomorphological variation suggested the involvement of temporal factors in disease progression. To investigate spatial and temporal pleomorphism in relation to viral replication activity, 42 autopsy brain samples were analyzed. Similar to a previous study in which demyelination foci were zoned in four autopsy cases [15], we classified demyelinated lesions into eight morphological types, ranging from the outermost, very early phase to the terminal stage of PML center lesions (Fig. 4). These PML morphological types 1–8 were observed sequentially from the outer border to the demyelinated center, especially in autopsies.

**Fig. 5:**
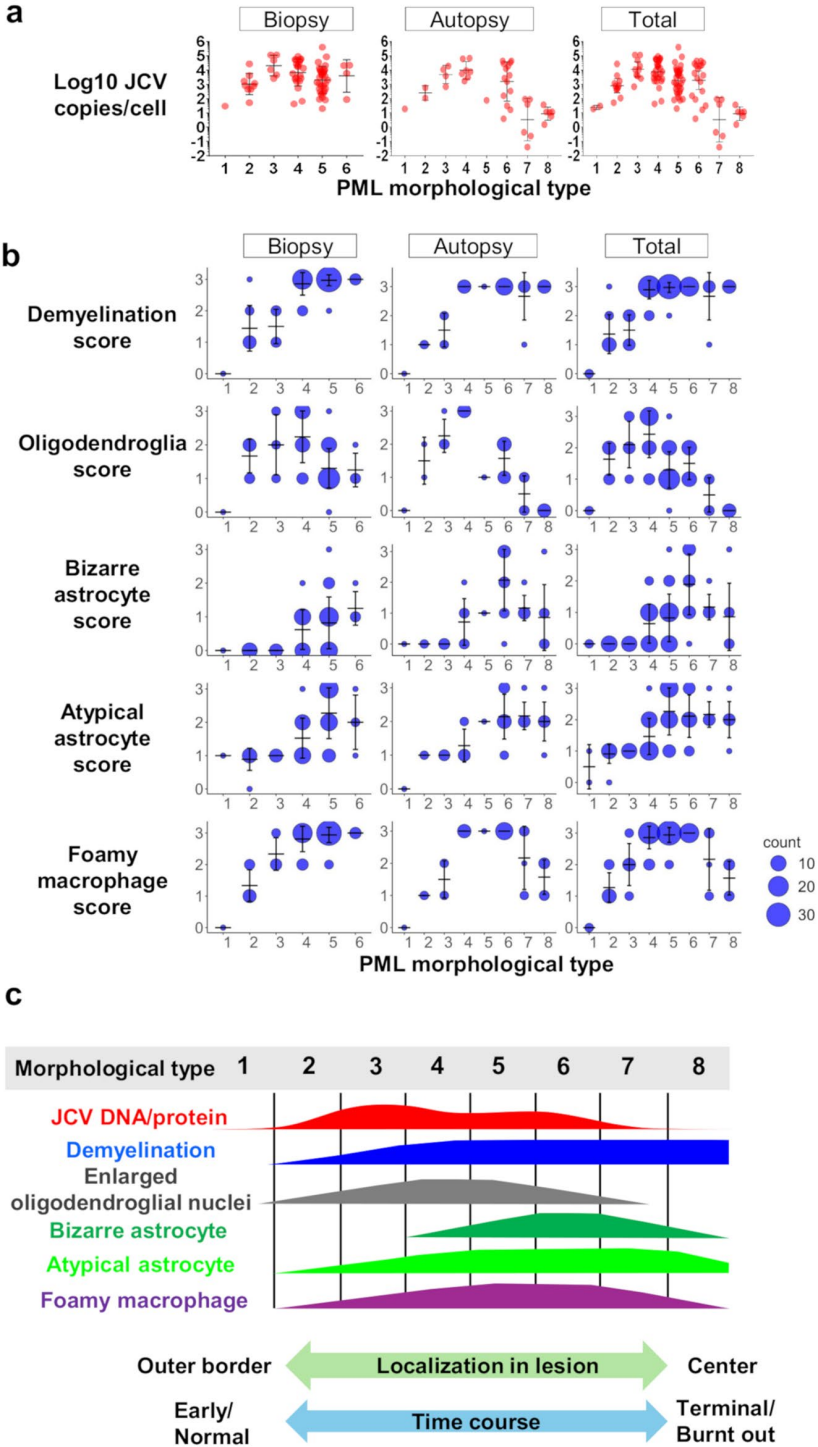
PML morphological types and JCV copies or histological feature scores. **a** PML morphological types and JCV copies in dot plots. Biopsy samples (*n* = 73), autopsy brains (*n* = 41), and total biopsy and autopsy brains (*n* = 114) were used. Geometric means with geometric SDs are also shown. **b** Bubble plots for PML morphological types and histological feature scores of demyelination, enlarged nucleated oligodendroglia, bizarre astrocytes, atypical astrocytes, and foamy macrophage infiltration. Biopsy (*n* = 74), autopsy (*n* = 42), and total samples (*n* = 116) were used. Means with standard deviations (SDs) are shown. The bubble sizes for counts are indicated at the bottom right. **c**. Schematic illustration of histopathological features with JCV copies and proteins across PML histomorphological types. The lower arrows indicate the lesion location and timing associated with each type

On the basis of each sample’s representative histomorphology, the 74 biopsy and 42 autopsy samples were classified into these eight morphological types. When a sample included more than one morphological type, it was assigned to the most dominant type observed in the same section. While all biopsy samples fell within Types 1–6, many autopsy samples contained Type 6 or later. Therefore, we combined biopsy and autopsy samples for a comprehensive analysis of lesion progression. In total, 116 samples classified by morphological type were first evaluated for viral copy numbers (Fig. 5a). Next, we assessed the classical histomorphological triad, atypical astrocytes, foamy macrophage scores, and PML morphological types (Fig. 5b). JCV copy numbers were highest in Types 3 and 4, with enlarged oligodendroglial nuclei most prominent in these types. Demyelination scores increased from Types 1 to 3 and plateaued after Type 4. Atypical astrocyte numbers increased from Types 1 to 4, peaking in Types 5 to 7. Bizarre astrocytes were rarely observed in Types 1 to 4 but increased from Type 4, peaking in Type 6, and then decreased in Types 7 and 8. Foamy macrophage infiltration begins in Type 2, peaks in Type 4, and plateaus before decreasing after Type 6. These morphological alterations, with tissue viral copies and proteins (described later), are summarized schematically in Fig. 5c.

**Fig. 6:**
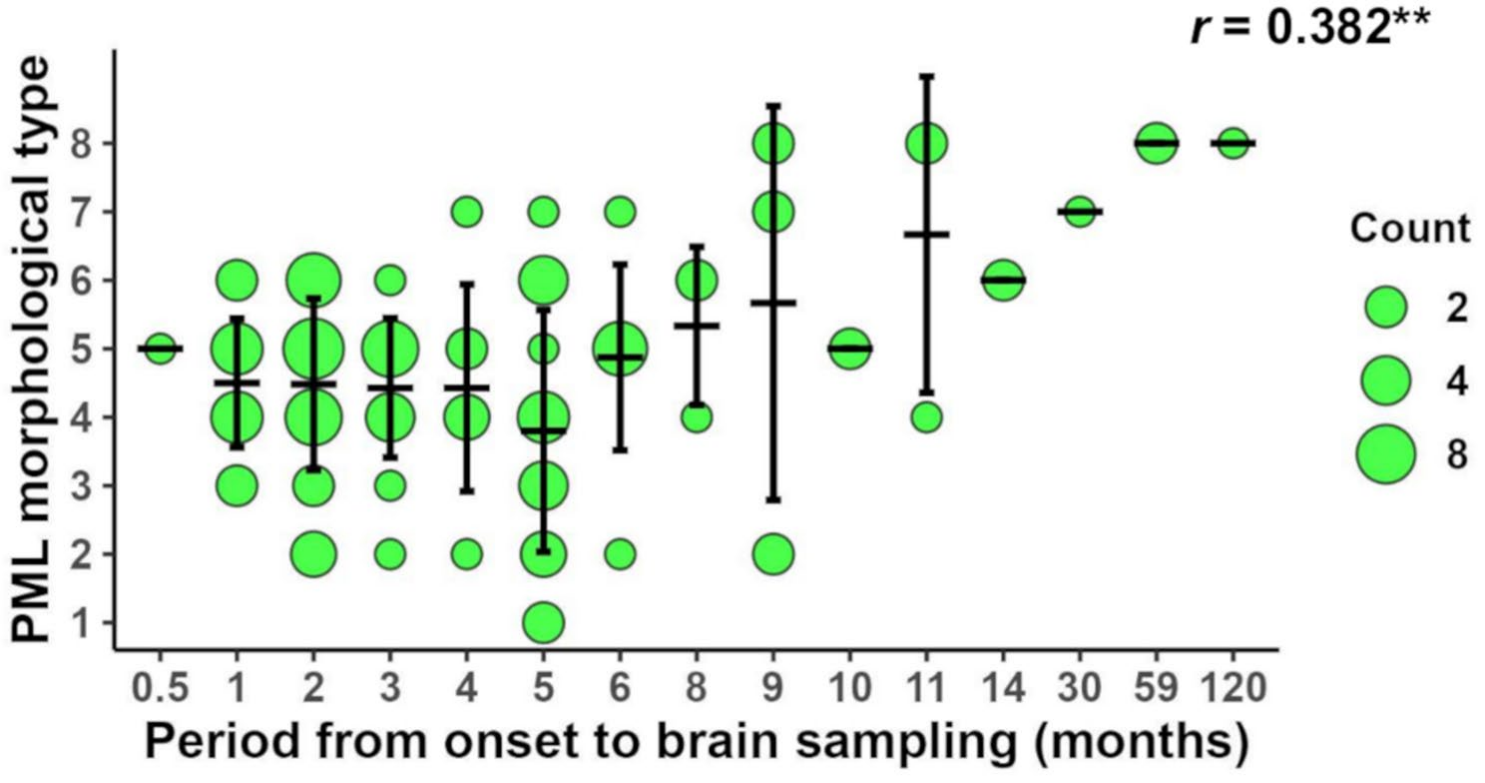
Relationships between the period from symptom onset to brain sampling and the representative PML morphological type of each sample (*n* = 111). Means with SDs are shown by horizontal and vertical lines. The bubble sizes for counts are indicated on the right of the panel. The Pearson product‒moment correlation coefficient (*r*) is shown at the top of the panel. **p < 0.01

Additionally, the time from symptom onset to brain sampling was analyzed against the most representative morphological type in each sample. Although many slides contained a range of morphological types, the dominant morphology indicated more active lesions in earlier cases and older demyelinated or “burnt-out” lesions in prolonged cases. Moreover, the period from symptom onset to brain sampling was significantly correlated with the number of morphological types (1–8) (Pearson *r* = 0.382, *R*² = 0.1463, *p* < 0.01), suggesting that the spatial variability of PML morphological types is correlated with temporal disease progression (Fig. 6).

### JCV protein expression and viral DNA loads

**Fig. 7:**
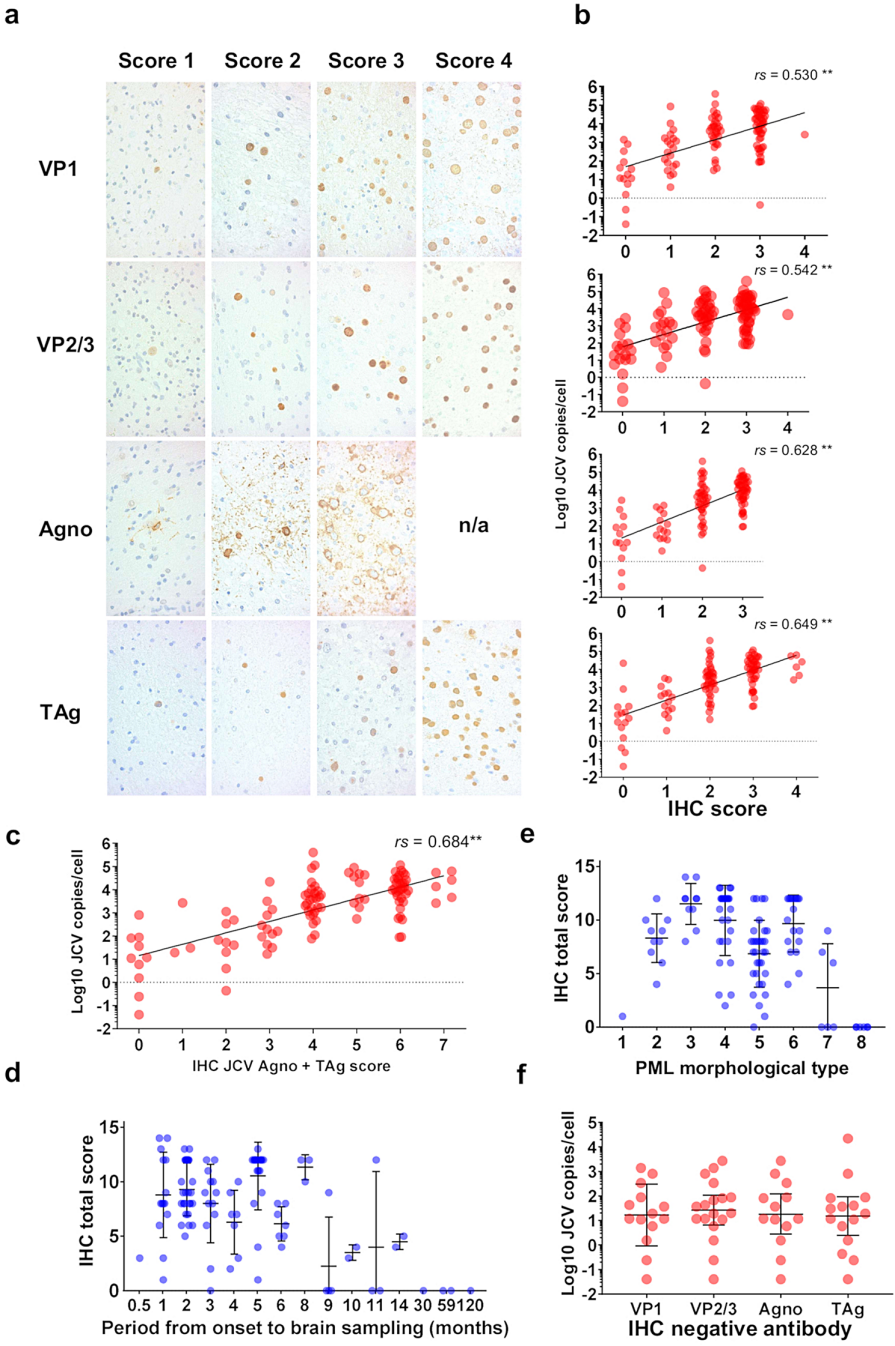
IHC scoring of PML brains and analysis of viral copy numbers and disease progression. **a** Scoring of IHC in PML brain samples. No specimen reached a score of 4 for agnoprotein (Agno). **b** IHC scores and JCV copies. VP1 (*n* = 114), VP2/3 (*n* = 113), Agno (*n* = 113), and TAg (*n* = 114). **c** IHC scores of Agno + TAg and JCV copies (*n* = 112). **d** Relationship between the period from symptom onset to brain sampling and total IHC (VP1 + VP2/3 + Agno + TAg) scores (*n* = 106). The means with SDs are shown. **e** PML morphological types and total IHC scores (*n* = 111). The means with SDs are shown. **f** IHC score-negative cases and JCV copies in the brain. Geometric means with geometric SDs are shown. Fitted lines are shown with Spearman’s correlation coefficient (*rs*) in **b** and **c**. **p < 0.01

To examine the relationship between the number of IHC-positive cells and viral replication activity, the IHC slides were scored on a scale from 0 to 4 (Fig. 7a) and compared with the viral loads in the tissue. The sensitivity of the antibodies was 87.1% for JCV VP1, 83.5% for JCV VP2/3, 87.8% for JCV agnoprotein, and 86.1% for TAg. All the IHC scores for the viral proteins were significantly positively correlated with the number of viral DNA copies (Fig. 7b), with TAg displaying the highest correlation (*rs* = 0.649, *p* < 0.01). Among the antibodies, the combination of IHC for JCV agnoprotein and TAg had the strongest correlation with viral DNA copy number (*rs* = 0.684, *p* < 0.01) (Fig. 7c). The detailed IHC scores and their correlations with viral DNA copy numbers are presented in Table 4. Additionally, no significant differences in the IHC scores were observed among the hematologic malignancy, autoimmune disease, and AIDS groups (Suppl. Fig. S3).

**Table 4:** Correlation of IHC scores and JCV copies

IHC	Spearman *r* (rs)	*p* value	Sample number
VP1	0.530	< 0.01	114
VP2/3	0.542	< 0.01	113
Agnoprotein	0.628	< 0.01	113
TAg	0.649	< 0.01	114
VP1 + VP2/3	0.536	< 0.01	112
VP1 + Agno	0.592	< 0.01	112
VP1 + TAg	0.643	< 0.01	113
VP2/3 + Agno	0.591	< 0.01	112
VP2/3 + TAg	0.631	< 0.01	112
Agno + TAg	0.684	< 0.01	112
VP1 + VP2/3 + Agno	0.577	< 0.01	111
VP1 + VP2/3 + TAg	0.613	< 0.01	111
VP1 + Agno + TAg	0.655	< 0.01	111
VP2/3 + Agno + TAg	0.651	< 0.01	111
VP1 + VP2/3 + Agno + TAg	0.630	< 0.01	110

Total IHC scores, calculated as the sum of IHC scores for VP1, VP2/3, agnoprotein, and TAg, were further analyzed in relation to the period from symptom onset to brain sampling and PML morphological types. Over time, total IHC scores decreased, becoming almost negative in prolonged cases (Fig. 7d). In terms of lesion type classification, total IHC scores increased from the outer early lesion to the demyelinated area, peaking at Type 3, where many enlarged oligodendroglial nuclei were observed just outside the demyelination border. The scores gradually decreased toward the center, becoming negative in the final stages (Fig. 7e).

Furthermore, the tissue JCV DNA loads in IHC-negative samples ranged from 15.1 to 26.3 copies per cell in geometric means, with no significant differences observed between the antibodies (Fig. 7f).

## Discussion

In this study, we analyzed 117 brain samples from 91 pathologically confirmed PML patients. Histomorphological features were scored quantitatively (Fig. 1a), and lesions were sequentially classified from early outer areas to advanced demyelination centers (Fig. 4). The pleomorphic alterations in the brain tissue were reconstructed and expanded upon previous studies, incorporating further analysis through histological scoring, PCR, and IHC [14, 15]. PML brain samples exhibited significant histomorphological variability, with each morphological type displaying distinct features. Real-time PCR for JCV DNA showed 100% sensitivity, and viral genome quantities were determined for each sample (Fig. 2a). As a result, histological features, disease progression, and viral activity were closely interconnected (Figs. 2, 3 and 5a, and Table 3). IHC for viral proteins showed a sensitivity between 83.5% and 87.8%, with significant correlations with viral loads (Fig. 7a-c, and Table 4). The histological scores, viral copy numbers, and IHC scores across the classified morphological types demonstrated spatial and temporal changes in PML tissue, providing pathologists with valuable quantitative data to interpret specimens in the context of viral activity and disease progression.

Initially, we examined the frequency of classical histomorphological features in PML brain samples. We found that not all the samples exhibited the complete PML triad. Autopsy samples, typically from advanced cases, often lack enlarged oligodendroglial nuclei, whereas biopsy samples from earlier stages contain fewer bizarre astrocytes (Fig. 1b). These findings partially align with previous observations that virus-laden oligodendrocytes are hallmarks of early lesions at demyelination borders and that bizarre astrocytes are more prominent in larger demyelination centers [16]. Since most autopsies are performed at the final stage of the disease, it is reasonable that many samples demonstrated advanced stages, including cases of “burnt-out” PML, where enlarged oligodendroglial nuclei were absent [16–18] (Figs. 4 and 5b). Conversely, biopsy materials, which are typically small and collected during earlier phases for clinical diagnosis, present fewer bizarre astrocytes, which are more commonly found in large demyelinating lesions or in later stages, such as Type 6 in our classification [16] (Figs. 4 and 5b). Given that PML histology evolves with lesion distribution and disease progression, a wide range of morphological variations can be observed [14, 15]. Therefore, the presence of the complete triad should not be expected in all samples: enlarged oligodendroglial nuclei are rare in autopsy samples from prolonged cases, and bizarre astrocytes are less common in biopsy samples from early lesions.

Second, we assessed viral activity in each brain tissue sample via quantitative PCR to detect JCV DNA. The combination of morphological and PCR analyses allows pathologists to provide a more integrated interpretation of histological features alongside pathogen activity. This study is the first to report viral copy numbers, including percentile values, in a large series of PML brain tissues. Compared with the autopsy samples, the biopsy samples presented higher viral copy numbers (Fig. 2a; Table 1), likely because the biopsy samples captured more active, early-phase lesions where the virus was replicating and proliferating more intensely than the autopsy samples did (Fig. 5a).

The combination of histological features and viral copy number analysis revealed that enlarged oligodendroglial nuclei had the strongest correlation with viral activity (Fig. 3; Table 3). This is expected, as oligodendrocytes are the primary target of JC viral lytic infection, leading to demyelination in the PML brain [32, 33]. Thus, enlarged oligodendroglial nuclei serve as the most reliable morphological indicator of viral activity in PML. Foamy macrophage infiltration was also correlated with the viral load (Fig. 3; Table 3), but since macrophages remain prominent even in later phases when the viral load decreases (Fig. 5b), their correlation was weaker. Other features, such as demyelination, bizarre astrocytes, and atypical astrocytes, did not significantly correlate with the number of viral copies (Fig. 3; Table 3). In earlier lesions, demyelination progressed alongside foamy macrophage infiltration (Fig. 5b), but bizarre astrocytes—although some were IHC positive—were not directly linked to viral infection or activity (Fig. 3; Table 3) [14, 16, 25].

Our analysis, which combines morphological classification with viral load data, suggests that a novel qualitative and quantitative pathological approach could guide precision therapy. The classification system, which is based on typical morphological features from the outer border to the demyelinated center, allowed us to describe the spatial and temporal evolution of PML pathology (Fig. 4). We demonstrated that viral copy numbers gradually increased to Type 3 and 4 lesions and then decreased in later stages (Figs. 4 and 5a). Early samples, especially those taken within 6 months of symptom onset, had significantly greater viral loads than later samples did (Fig. 2a-b). These findings highlight the importance of brain biopsies that target active lesions at the demyelination border, particularly within 6 months of onset, for accurate diagnosis and effective antiviral therapy. During this phase, JCV replication is most active in Type 3 and 4 lesions, making these regions ideal for biopsy and subsequent treatment planning. Conversely, biopsies taken in later phases may target more advanced or “burnt-out” lesions with reduced viral activity, limiting the effectiveness of antiviral therapy (Figs. 4 and 5). Although PCR is sensitive enough to detect JCV even in protracted cases [18], earlier cases with more copies provide more valuable diagnostic information and are more responsive to treatment. Therefore, brain biopsy at the demyelination border during the appropriate phase, coupled with quantitative PCR evaluation of viral activity, offers pathologists and clinicians critical insights for diagnosis and treatment strategies.

We developed an IHC scoring system to quantify what is traditionally a qualitative method (Fig. 7a), demonstrating that all viral protein antibodies showed a significant positive correlation with JCV copies in brain tissue. The combination of antibodies against JCV agnoprotein and TAg had the strongest correlation, with TAg alone also highly correlated with the viral load (Fig. 7b-c; Table 4). While TAg antibodies are commonly used for diagnosing polyomavirus infections, including PML, issues with sensitivity in FFPE samples and nonspecific staining in certain brain tumors and lymphomas suggest that combining antibodies is a more reliable approach [3, 34–37]. Agnoprotein, which localizes to cytoplasmic processes, provides broader IHC signals [38, 39]. Given that other antibodies primarily show nuclear staining (although VP1 can also be positive in the cytoplasm), the broad cytoplasmic signal of agnoprotein, which is easily recognized even apart from the nuclei, is particularly useful for diagnosis in small samples where other antibodies may not produce clear signals [2, 19, 30].

Our IHC scoring and morphological type analysis revealed higher scores in earlier lesions (Fig. 7d-e), which correlated with higher viral copy numbers (Figs. 2b and 5a). IHC-negative cases generally had fewer viral copies, suggesting that the threshold for IHC positivity corresponds to approximately 10 copies per cell in quantitative PCR (Fig. 7f). IHC-negative but PCR-positive cases typically fall into three categories: small biopsy samples lacking the classical triad, inflammatory cases or immune reconstitution inflammatory syndrome (IRIS), and “burnt-out” PML. In small biopsy samples without the triad [19, 40], agnoprotein IHC and PCR are particularly valuable. In inflammatory or IRIS cases, lymphocytic infiltration and fewer infected oligodendrocytes contribute to lower viral loads [3, 41, 42]. “Burnt-out” cases were negative for IHC but positive for PCR (Figs. 5a and 7d-e) [18], which is consistent with previous findings that viral DNA can still be detected in such lesions via in situ hybridization (ISH) [16]. Furthermore, ISH targeting JCV-encoded microRNA (miRNA) has shown comparable sensitivity to that of IHC and can be valuable even for past autopsy samples [43]. Since miRNAs are small nucleotide molecules that remain well conserved in FFPE samples, future studies utilizing ISH for viral miRNAs could be particularly useful, especially in IHC-negative cases [44–46].

This study has several limitations that should be considered when interpreting the findings. First, while JCV genomic variants are known to significantly influence viral replication and CNS tropism, most nucleic acid analyses in this study were performed using FFPE samples. Due to the degradation of nucleic acids in FFPE tissues, detailed sequencing analysis of viral variants for each case was not feasible, limiting our ability to fully elucidate the pathogenetic mechanisms driving disease progression. Second, nearly all brain tissue samples analyzed in this study were obtained for diagnostic purposes prior to the initiation of treatment. Consequently, we were unable to assess tissue changes following the administration of disease-modifying therapies. Third, the patient backgrounds in this study included a variety of immunosuppressive conditions, such as hematologic malignancies, AIDS, and autoimmune diseases, with some cases exhibiting inflammatory features in the tissue samples. However, this study did not aim to simultaneously evaluate inflammation and immune responses (innate or adaptive) in PML cases with inflammatory components. Further research is needed to comprehensively analyze the impact of inflammation on disease pathology and viral dynamics. Fourth, while this study analyzed PML cases across various stages of disease progression, most analyses did not separate patients with early-stage disease from those with long-standing disease. This approach was chosen to assess PML lesions as a continuous temporal and spatial process. However, we acknowledge that dividing the data into early-stage versus late-stage cases could provide additional insights (Fig. 2a). A comparative analysis between active disease and long-term survivors would require a different study design and is beyond the scope of this study.

Finally, with respect to underlying diseases, only AIDS-associated PML showed more severe demyelination (Suppl. Fig. S1), which is partially consistent with previous studies reporting increased tissue damage in these patients [16, 25]. However, no significant differences in other morphological features or viral copies were detected between AIDS-associated and non-AIDS PML patients (Fig. 2a and Suppl. Fig. S1). This may reflect the impact of modern antiretroviral therapy on AIDS-associated PML, which differs from classical PML cases [16]. IHC scores also showed no significant differences across underlying diseases (Suppl. Fig. S3), indicating that histopathological variations are likely driven by spatial and temporal factors rather than underlying conditions.

## Conclusions

Our study highlights the histological diversity of PML, which is driven by the spatial and temporal progression of the disease. Quantitative PCR revealed strong correlations between viral activity and IHC staining, with the highest viral loads found at the demyelination border, making this the ideal biopsy target. Since early-stage PML may lack the full histological triad, sampling from active demyelination borders improves diagnostic sensitivity. While we did not assess treatment responses, our findings provide a basis for stratifying patients by disease stage, potentially guiding personalized treatment approaches. Future integration of our classification with molecular biomarkers and imaging could further refine precision medicine strategies for PML.

## Electronic supplementary material

Below is the link to the electronic supplementary material.

Supplementary Material 1

## Data Availability

Data is provided within the manuscript or supplementary information files.

## Abbreviations

AIDS: Acquired Immunodeficiency Syndrome
CSF: Cerebrospinal Fluid
DNA: Deoxyribonucleic Acid
FFPE: Formalin-Fixed Paraffin-Embedded
HE: Hematoxylin and Eosin
HPF: High-Power Field
IHC: Immunohistochemistry
ISH: In Situ Hybridization
JCV: JC Polyomavirus
LPF: Low-Power Field
miRNA: MicroRNA
MRI: Magnetic Resonance Imaging
NIID: National Institute of Infectious Diseases
PML: Progressive Multifocal Leukoencephalopathy
PCR: Polymerase Chain Reaction
RNA: Ribonucleic Acid
SV40: Simian Virus 40
TAg: Large Tumor Antigen
VP1: Viral Protein 1
