# Supplementary material for: Histomorphological variations in progressive multifocal leukoencephalopathy correlated with JCV replication in brain lesions: insights from 91 patients
Source: Acta Neuropathol Commun. 2025 May 19;13:106. doi: 10.1186/s40478-025-02027-7 (PMC12087142; doi:10.1186/s40478-025-02027-7)
Supplement: Supplementary file 1 — Supplementary Material 1 [file 40478_2025_2027_MOESM1_ESM.pdf]

Supplementary information

Histomorphological Variations in Progressive Multifocal Leukoencephalopathy Correlated with JCV Replication in Brain Lesions: Insights from 91 Patients

Acta Neuropathologica Communications

Kenta Takahashi, Yuko Sato, Hideki Hasegawa, Harutaka Katano*, and Tadaki Suzuki*

*Corresponding authors: katano@niid.go.jp, tksuzuki@niid.go.jp

Supplementary Fig. S1-S3

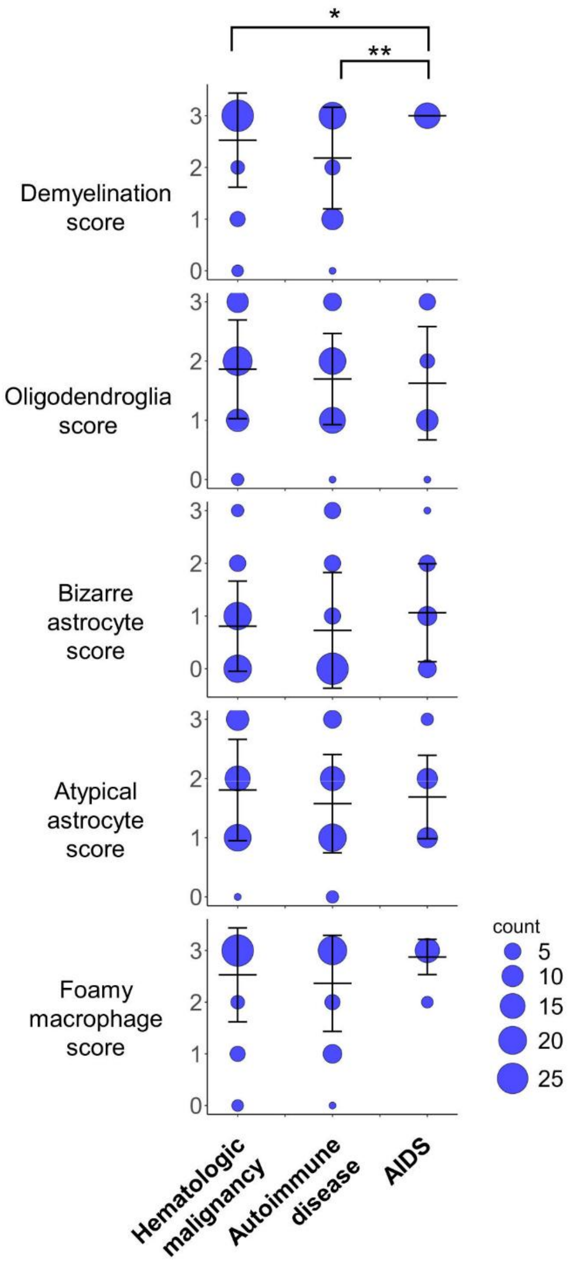

Supplementary Fig. S1. Underlying diseases and PML morphologic scores.
The underlying diseases analyzed include hematologic malignancies, autoimmune diseases, and AIDS, with sample sizes of n = 36, 33, and 16, respectively. Long horizontal bars represent the means, and error bars indicate standard deviations (SDs). Asterisks denote significant differences between groups ($*p < 0.05$; $**p < 0.01$).

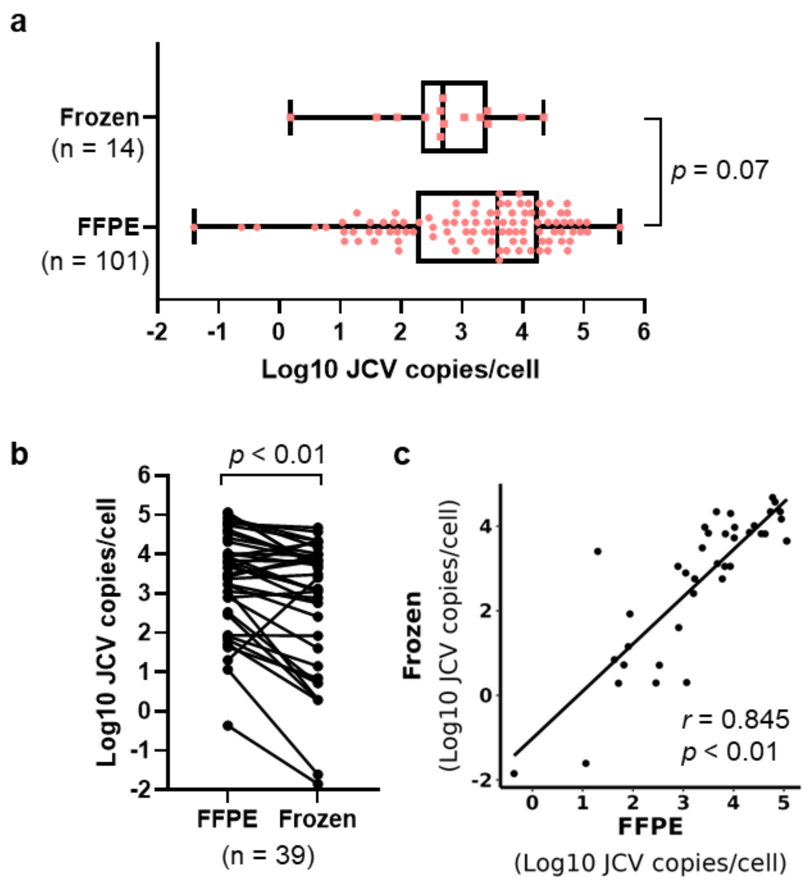

Supplementary Fig. S2. Quantification of JCV DNA in PML brain tissues using FFPE and frozen samples.

a JCV copies in PML brain tissues (n = 115). FFPE and frozen samples (n = 101 and 14) were used. Boxes show medians and 25% and 75% percentiles, and whiskers indicate the range from minimum to maximum values. **b** Comparison of JCV copies in FFPE and frozen samples using 39 paired samples with both FFPE and frozen tissues available. P value was calculated with paired t-test. **c** Relationship between the results from FFPE and frozen samples of JCV copies in the 39 paired samples with both FFPE and frozen tissues available. The x- and y-axes represent $\log_{10}$ JCV copies per cell. A fitted line is shown, along with the Pearson correlation coefficient (*r*).

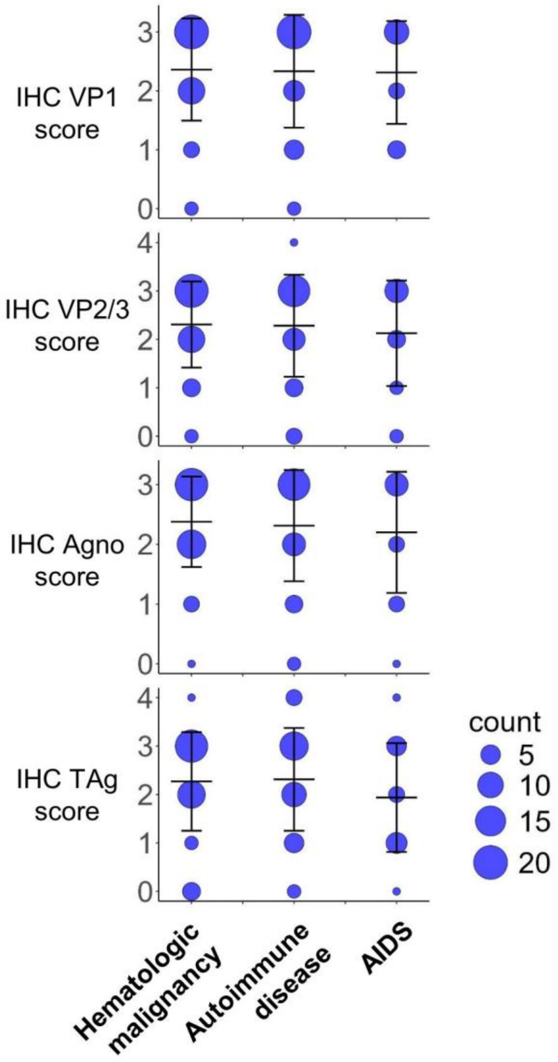

Supplementary Fig. S3. Underlying diseases and IHC scores.

The underlying diseases (hematologic malignancies, autoimmune diseases, and AIDS) were analyzed using the same sample set as in Supplementary Fig. S1. Immunohistochemistry (IHC) was performed for VP1 (n = 85), VP2/3 (n = 84), agnoprotein (Agno) (n = 84), and TAg (n = 85). For details on the IHC scoring system, refer to the main text. Long horizontal bars represent the means, and error bars indicate standard deviations (SDs).
